# Extracellular Vesicles: Evolving Factors in Stem Cell Biology

**DOI:** 10.1155/2016/1073140

**Published:** 2015-11-16

**Authors:** Muhammad Nawaz, Farah Fatima, Krishna C. Vallabhaneni, Patrice Penfornis, Hadi Valadi, Karin Ekström, Sharad Kholia, Jason D. Whitt, Joseph D. Fernandes, Radhika Pochampally, Jeremy A. Squire, Giovanni Camussi

**Affiliations:** ^1^Department of Pathology and Forensic Medicine, Faculty of Medicine Ribeirao Preto, University of Sao Paulo, Avenue Bandeirantes, 3900 Ribeirao Preto, SP, Brazil; ^2^Department of Rheumatology and Inflammation Research, University of Gothenburg, Box 480, 405 30 Gothenburg, Sweden; ^3^Cancer Institute, University of Mississippi Medical Center, Jackson, MS 39216, USA; ^4^BIOMATCELL VINN Excellence Center of Biomaterials and Cell Therapy, 40530 Gothenburg, Sweden; ^5^Department of Biomaterials, University of Gothenburg, Box 412, 405 30 Gothenburg, Sweden; ^6^Cellular and Molecular Immunology Research Centre, School of Human Sciences, London Metropolitan University, London 166-220, UK; ^7^Department of Medical Sciences and Molecular Biotechnology Center, University of Torino, Corso Dogliotti 14, 10126 Torino, Italy

## Abstract

Stem cells are proposed to continuously secrete trophic factors that potentially serve as mediators of autocrine and paracrine activities, associated with reprogramming of the tumor microenvironment, tissue regeneration, and repair. Hitherto, significant efforts have been made to understand the level of underlying paracrine activities influenced by stem cell secreted trophic factors, as little is known about these interactions. Recent findings, however, elucidate this role by reporting the effects of stem cell derived extracellular vesicles (EVs) that mimic the phenotypes of the cells from which they originate. Exchange of genetic information utilizing persistent bidirectional communication mediated by stem cell-EVs could regulate stemness, self-renewal, and differentiation in stem cells and their subpopulations. This review therefore discusses stem cell-EVs as evolving communication factors in stem cell biology, focusing on how they regulate cell fates by inducing persistent and prolonged genetic reprogramming of resident cells in a paracrine fashion. In addition, we address the role of stem cell-secreted vesicles in shaping the tumor microenvironment and immunomodulation and in their ability to stimulate endogenous repair processes during tissue damage. Collectively, these functions ensure an enormous potential for future therapies.

## 1. Introduction

Stem cell technology has recently attracted considerable attention in translational medicine due to the potential that these cells possess in terms of tissue regeneration and repair and as drug delivery tools for which existing therapeutic strategies pose enduring challenges. In recent years, the fields of regenerative and translational medicine have proven to be very attractive owing to the discovery of novel cellular and noncellular therapeutic platforms for tissue repairs and cancer treatments.

This review mainly engages studies carried out on the two major types of stem cell lines: embryonic stem cells (ESCs) and mesenchymal stem cells (MSCs). Nevertheless, several other types of stem cells closely related to their tissue of origin (e.g., adipose stem cells, cancer stem cells) have also been reported.

ESCs are pluripotent cells with the ability to differentiate into cells from any of the three germ layers: mesoderm, endoderm, and ectoderm. They have the capability to self-renew and proliferate limitlessly, but their application in research and therapy has been limited due to ethical concerns on availability and the risk of forming teratomas.

In the last two decades, more attention has been diverted towards MSCs as they are easily obtainable and show therapeutic promise. MSCs are a nonhematopoietic, heterogeneous population of plastic-adherent cells that exhibit a fibroblast-like morphology. They form distinct colonies when seeded at clonal densities, and their heterogeneity is distinguished through morphological differences, rate of proliferation, and their ability to differentiate [1]. According to the current nomenclature, human MSCs can be identified through their positivity for cell surface markers such as CD73, CD90, and CD105 and the lack of expression of hematopoietic markers such as CD11b or CD34, CD45, CD79 or CD19, and HLA-DR [2]. Furthermore, they must have the ability to differentiate into osteoblasts, chondrocytes, and adipocytes* in vitro* [2]. The biological effects of MSCs depend largely on their ability to secrete trophic factors that stimulate tissue-intrinsic progenitor cell phenotypes [3]. These factors include growth factors, miRNAs, and small vesicles that not only potentially affect the differentiation and regenerative abilities of MSCs but also play a critical role in mediating crosstalk to local and distant tissues through which stem cell populations maintain a stable coexistence [4].

Recent evidence shows that stem cells secrete small vesicles into the extracellular milieu, known as extracellular vesicles (EVs). EVs are submicron vesicles, which based on their size, origin, morphology, and mode of release can be categorized into exosomes (40–200 nm), microvesicles (50–1000 nm), apoptotic bodies (50–5000 nm), or Golgi vesicles (reviewed in [5]). EVs are secreted by a multitude of cell types into various body fluids [6] and can be isolated via several conventional as well as high throughput technologies [5]. They are known to carry a repertoire of mRNAs, miRNAs, DNA, proteins, and lipids that can be transferred to neighboring cells, modifying their phenotype as well as the microenvironment [7, 8]. The molecular signatures of EVs are selective to each cell/tissue type, which makes them ideal source for clinical applications [5].

The biogenesis and secretion of EVs from biologically active cells are a stimulus dependent event that is arising as a result of tumor progression or repair processes. A well-studied process of formation of exosomes is by the fusion of the multivesicular endosome with plasma membrane and release by the process of exocytosis. Conversely, microvesicles are less well characterized in comparison to exosomes and are produced as a result of membrane budding processes and detachment of spherical bodies from discriminatory regions of the plasma membrane (for mechanisms, see [5]). Recently, Golgi vesicles were highlighted, considering them a part of the extracellular vesicle populations as separate entities. Like other vesicles, they may reflect distinct disease states [5], and their role could be hypothesized for underappreciated effectors in stem cell context.

Interestingly, it has been shown recently that MSC exosomes are derived from endocytosed lipid-raft microdomains [9]. Arguably, the knowledge about the mechanisms of biogenesis and origin of EV populations from stem cells could enhance our understanding of the functional relevance and help acquire therapeutic possibilities using stem cell-EVs.

## 2. Intercellular Communication and Transfer of Biological Information

Cells use several means of communication for the exchange of materials and the transfer of information in order to maintain tissue homeostasis, cell development, repair, and survival. Intercellular communication is therefore considered one of the most important regulatory mechanisms for cell growth, differentiation, and tissue remodeling. This intercellular crosstalk may occur through direct receptor-mediated interaction between cells, or through cellular junctions (i.e., cell fusion). Certain molecules, specific transmembrane proteins, and cell adhesion molecules such as integrins, tetraspanins, and cadherins are known to promote receptor-mediated cellular interactions that are critical in the formation of biological patterns as well as determining cell fates [10, 11]. On the other hand, the direct coupling of the cytoplasm of two cells through gap junctions (GJs) has also been reported to play an essential role in maintaining tissue homeostasis, development, and stem cell differentiation [12, 13].

It has been proposed that normal human adult stem cells do not express gap junctional proteins (i.e., connexins) and do not appear to have gap junctional intercellular communication. However, their differentiated, nonstem cell derivatives do express connexins and therefore utilize GJs for intercellular communication in order to differentiate [14]. Remarkably, bone marrow derived MSCs have been reported to differentiate into cells with a cardiac phenotype in response to signals from neighboring myocytes, as a result of gap junctional intercellular communication [15]. Arguably, the crucial role that these interactions play in the maintenance of tissue integrity in a plethora of different cell types such as endothelial cells, fibroblasts, adipocytes, cardiomyocytes, and neurons is of critical translational importance.

### 2.1. Tunneling Nanotubes and Intercellular Communication

Recently, a novel mechanism for cellular communication involving long distance intercellular connections called tunneling nanotubes (TNTs) has come to light [16]. TNTs are actin-based cytoplasmic extensions that not only facilitate direct communication between distant cells and intercellular trafficking [17] but also are associated with the transfer of biomolecular cargos such as organelles, Golgi vesicles, plasma membrane components, and even pathogens [18–20]. These functions of TNTs have therefore implicated them in the promotion of various physiological (tissue development and regeneration [21]) as well as pathological processes such as mesothelioma [22].

For instance, extensive spontaneous intercellular exchange of cytosolic materials and organelles, between primary human proximal tubular epithelial cells, was recently reported [23]. Interestingly, this exchange was alleviated significantly on inhibiting TNT genesis, therefore implying the importance of TNTs in renal physiology [23]. Surprisingly, the direct transfer of genetic material between tumor and stromal cells has also been identified to be mediated through TNTs, exclusive of other forms of cell-cell communication, therefore suggesting a role of TNTs in tissue remodeling as well [24]. A recent study described the nanotubular connections and tubule fragments in multipotent MSCs from human arteries, demonstrating that they interact constitutively through an articulate and dynamic tubule network allowing long-range cell-to-cell communication [25]. This therefore suggests that one mechanism by which MSCs exhibit their protective effects on the heart may be through tubular communication.

### 2.2. Cell-Cell Communication through Paracrine Secreted Factors

Diffusion of autocrine and paracrine signaling molecules enables cells to communicate in the absence of physical contact. The beneficial effects of stem cells are therefore restricted not only to cell-to-cell contact alone, but also to their transient paracrine actions through the release of a combination of trophic factors including cytokines, chemokines, and growth factors [26–28]. These factors have been shown to have various functional properties such as being antiapoptotic, immunomodulatory, and angiogenic, playing a role in cell-mobilization, evoking responses from resident cells in the extracellular environment, and facilitating collateral remodeling [29]. Through receptor-mediated interactions, these paracrine mechanisms potentially facilitate the homing of stem cells to sites of tissue injury, therefore allowing recipient cells to interact with stem cell factors that may influence the regulation of differentiation and anti-inflammatory responses [26].

Cells could also communicate through secreted extracellular RNAs, which, once transferred from one cell to another, may modulate the phenotype and function of the recipient cells. Furthermore, the exchange of extracellular RNAs between resident cells and MSCs has been implicated in the modulation of cell fate [30]. These extracellular RNAs are secreted, vesicle-encapsulated or in association with proteins that confer protection from degrading enzymes and contribute towards the microenvironmental modulation of cell fate with other extracellular factors such as the extracellular matrix as well as the local biochemical and mechanical niches [31].

### 2.3. Extracellular Vesicles as Novel Means of Communication

Secreted EVs exhibit novel paracrine mechanisms mediating cell-to-cell communication through receptor-ligand mediated interactions and/or direct fusion with resident cells, resulting in the horizontal transfer of various proteins as well as genetic material (including mRNA [8, 32–34] and miRNA [34]). As paracrine factors, EVs have been reported to induce phenotypic changes in recipient cells and also generate a functional link between stem cells and tissues under various physiological and pathological circumstances (reviewed in [33]).

Recently, it has been demonstrated that EVs released by human adipocytes or adipose tissue-explants are involved in a reciprocal proinflammatory loop between adipocytes and macrophages in a paracrine fashion, with the possibility to aggravate insulin resistance locally or systemically [35]. Moreover, EVs released from osteoblasts serve as a mechanism of communication between osteoblasts and osteoclasts through receptor-ligand interactions to facilitate osteoclast formation that represent a novel mechanism of bone remodeling [36].

Although stem cells have been considered safe for available therapeutic strategies, there are still potential risks and complications that may arise such as culture-induced senescence, immune-mediated rejection, genetic instability, loss of functional properties, and consequent malignant transformations. In this perspective, there is a prospect that stem cell-EVs may overcome these limitations and therefore offer novel and safe cell-free therapeutic opportunities. However, their promise as real clinical applications still awaits further investigations yet to be fully realized.

## 3. Extracellular Vesicles Mimic Stemlike Phenotypes

Recent data from various different studies have shown stem cell-EVs to traffic stem cell associated transcription factors such as Nanog, Oct-4, HoxB4, and Rex-1 operating at the level of pluripotent stem cells [37]. On top of that, they have also been shown to express MSC markers such as CD105 [38], prominin-1/CD133 [39–41], and KIT [42]. More direct effectors of the stem cell phenotype such as WNT [43, 44], *β*-catenin [45], and Hedgehog [46] have also been identified on stem cell-EVs together with several other components that may be considered potential factors in stem cell biology (Table 1). Furthermore, ESC-EVs can be harvested from exponentially growing ESCs, therefore suggesting that the mechanism of EV release from ESCs is inherent [37].

Emerging evidence reveals that secreted vesicles from stem cells could mimic a spectrum of stemlike phenotypes. Ratajczak and coworkers provided the first evidence that EVs released from stem cells might modulate the phenotype of recipient cells [37]. In this study, they reported that EVs released from ESCs sustained the maintenance of hematopoietic stem/progenitor cell stemness and multipotency by delivering specific proteins and mRNA, subsequently upregulating the expression of early pluripotent and hematopoietic stem cell genes [37]. A following study further confirmed this by reporting that mRNA-carrying EVs from endothelial progenitors induced a proangiogenic phenotype in quiescent endothelial cells [47]. Moreover, it was recently revealed that EV-mediated mRNA delivery into marrow cells could introduce tissue-specific changes through the induction of transcription in recipient cells [48], further indicating that mRNAs present in stem cell-EVs are functional and have regulatory effects.

Several studies have revealed that the key biological features of stem cells, such as self-renewal, differentiation, and maturation, could be mimicked by stem cell associated EVs [49–53]. Furthermore, stem cell-EVs have been reported to coordinate physiological self-regenerative and repair processes after tissue injury in a paracrine manner. For instance, EVs released from MSCs (MSC-EVs) activate repair processes by transferring miRNAs [54] and promoting angiogenesis [55, 56], therefore suggesting EVs as functional extensions of stem cells.

Interplay between stem cells and EVs reveals that EVs could transmit multipotential traits and represent several features of MSCs, such as multilineage differentiation, self-renewal, and transcriptional regulation, therefore, inferring their role as evolving factors in stem cell biology.

## 4. Stem Cell-EVs Contribute to the Cell-Fate Determination

**Figure 1:**
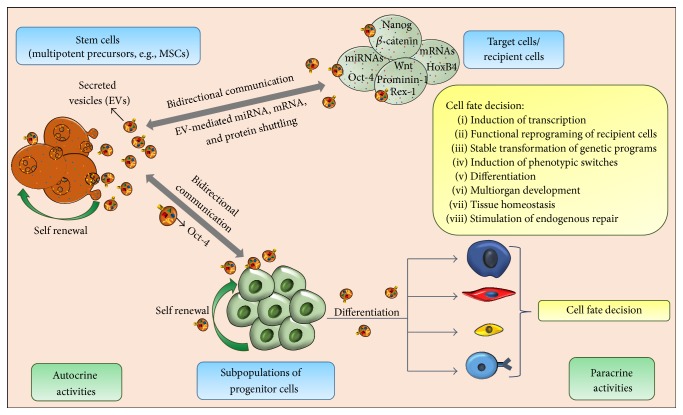
The role played by stem cell-derived EVs in the determination of cell fate. Stem cells use EVs to transfer miRNAs and stem cell effectors in recipient cells, which target the regulatory networks and induce persistent genetic transformation and phenotypic switching of resident cells towards cell-fate determination.

There is sufficient evidence to postulate that EVs carry biological messages from parent cells that interact with recipient cells and influence their normal physiology and therefore their overall fate [33]. In the context of stem cell biology, it is likely that the same principle applies (Figure 1), as biological content from stem cell-EVs has been shown to have the capability to influence and define cell fates of future populations of resident cells, potentially producing long lasting and stable transformation in genetic programs [48, 57]. Furthermore, Quesenberry et al. have also proposed that EV-mediated exchange of genetic information could be an integral element of the continuum model of stem cell biology, in which the differentiation decision of stem cells is conditioned by the cell cycle transit and environmental stimuli [58].

### 4.1. EV-Mediated miRNA Transport and Cell-Fate Determination

To sustain stemness, a set of transcriptional factors are required to efficiently reprogram and self-renew the pluripotency of recipient cells to enable them to differentiate into various cell types [59]. Indeed, miRNAs are capable of targeting and regulating the expression of stem cell associated transcriptional networks by which they can induce phenotypic transition [60, 61]. In this context, EVs contribute to such reprogramming and phenotypic switching by transferring miRNAs as the delivery of selective sequestered miRNAs in EVs to recipient cells has been reported [34].

For instance, EVs from bone marrow- (BM-) MSCs shuttle the selected pattern of miRNAs to recipient cells targeting genes involved in multiorgan development, cell survival, and differentiation [54]. Furthermore, ESC-EVs induce dedifferentiation and the expression of pluripotency factors in their target cells by selective transfer of mRNA and miRNA, which may initiate an early differentiation process by targeting early transcriptional factors [62]. It has also been reported that telocytes (TCs) secrete EVs loaded with miRNAs that could modulate stem cell fates through continuous posttranscriptional regulatory signals acting back and forth between TCs and stem cells [63]. Moreover, Let-7 miRNA family expressed from human embryonic MSCs has been shown to affect its downstream target hepatic nuclear factor 4 alpha (HNF4A), indicating the possible role of stem cells in differentiation processes through EV-mediated transfer of miRNAs [64]. In addition, several other miRNAs have been identified to potentially contribute to regulatory mechanisms in stem cell biology (Table 2). These reports therefore signify that stem cells may exhibit their biological effects through EV-mediated shuttle of miRNAs for the biological effects mediated by stem cells.

## 5. Stem Cell-EVs and Tumor Progression

The role of stem cells in tumor progression has been well documented [65]; however, the mechanisms by which cancer stem cell-derived EVs initiate and promote tumor progression are still uncertain. Recent evidence explains the interplay between stem progenitors and their secreted vesicles in tumor progression. For example, it has been reported that EVs positive for the stem cell marker prominin-1 participate in Wnt signaling and mediate prometastatic activity in melanoma cells [40]. In addition, EVs from mast cells express and shuttle KIT protein (mast/stem cell growth factor receptor), which in turn promotes tumor growth in recipient lung adenocarcinoma cells by activating the KIT-SCF signaling pathway [42]. EVs from BM-MSCs have also been shown to express higher levels of oncogenic proteins, cytokines, and adhesion molecules that could be transferred to multiple myeloma cells and modulate tumor growth* in vivo* [66]. Recently, Eirin et al. reported that adipose MSC (AT-MSC) derived EVs enriched with distinct mRNAs and miRNAs regulate various physiological processes such as angiogenesis, adipogenesis, apoptosis, and proteolysis in recipient cells [67]. Furthermore, EVs from BM-MSCs have been reported to not only transport tumor regulatory miRNAs, antiapoptotic proteins, and metabolites but also enhance the expression of vascular endothelial growth factor (VEGF) through the activation of extracellular signal-regulated kinase 1/2 (ERK1/2) pathway to promote growth of various tumors [68, 69]. In addition, gastric cancer derived MSC-EVs have been shown to deliver deregulated miRNAs to human gastric cancer cells and promote their proliferation and migration [70].

A subset of tumor-initiating cells expressing the MSC marker CD105 in human renal cell carcinoma release EVs that induce angiogenic phenotype in endothelial cells and promote the formation of a premetastatic niche [38, 71]. Furthermore, renal cell carcinoma-derived EVs induce persistent phenotypical changes in MSCs accompanied with enhanced expression of genes associated with cell migration, matrix remodeling, angiogenesis, and tumor growth [71].

To the contrary, various reports also suggest the antitumorigenic effects of EVs from multiple stem cell types. For instance, Bruno et al. have reported that BM-MSCs exert inhibitory effects on tumor growth through the release of EVs [72]. Furthermore, BM-MSC derived EV-mediated transfer of miRNAs from the bone marrow could promote dormancy in metastatic breast cancer cells [73]. In addition, various studies have reported the antiproliferative effects of MSC-EVs through various mechanisms, for instance, miRNA-mediated VEGF suppression in breast cancer cells [74] and the downregulation of phosphorylation of Akt kinase in bladder tumor cells [75]. Similarly, a study by Fonsato et al. shows that EV-mediated delivery of selected miRNAs from adult human liver stem cells (HLSC) exhibits inhibitory effects on hepatoma growth [76]. On the basis of this information and on various other reports, MSC derived EVs could therefore exert either anti- or protumorigenic effects depending on the tumor type and stage of development [77].

Glioma-associated stem cells (GASCs) produce substantial amounts of EVs that express and mediate glioma-stem cell characteristics [78–80]. For instance, a recent study reported that GASCs exhibit stem cell feature and anchorage-independent growth and are capable of sustaining malignant properties of both glioma cells and glioma-stem cells, mainly through the release of EVs [78]. Furthermore, the cargo (DNA, miRNA, transcripts, and proteins) from GASC derived EVs may influence lineage specific dynamics of the stem cell compartments [81].

MSC-EVs could also be engineered to play a potential role in cancer therapeutics as delivery vehicles. For instance, Munoz et al. successfully reported the delivery of functional anti-miR-9 to glioblastoma (GBM) cells through MSC-EVs, therefore increasing their level of chemosensitivity substantially [79]. In addition, EV-dependent phenotypes of stem cells such as neurosphere growth and endothelial tube formation were attenuated by loading miR-1 into GBM-EVs that were exposed to the glioblastoma microhabitat [80]. Furthermore, a recent study by Katakowski et al. observed that intratumor injection of miR-146 expressing MSC-EVs significantly inhibited glioma xenograft growth [82]. Likewise, Pascucci et al. also reported the potential ability of MSCs to package/incorporate and deliver active drugs such as paclitaxel through EVs to inhibit tumor progression [83]. These studies therefore indicate the potential capability and utility of stem cell-EVs as drug delivery mechanisms for cancer therapy.

### 5.1. Ways by Which Stem Cell-EVs Influence the Tumor Microenvironment

The principle properties of cancer stem cells (CSCs) are maintained by niches which are anatomically distinct regions within the tumor microenvironment [84]. The premetastatic niche plays a role in dormancy, relapse, and the development of metastasis. It has been hypothesized that EVs from CSCs may behave as metastasomes, helping the implementation of secondary lesions by transmission of the metastatic phenotype via EV-borne tumor RNA signatures to the target organ [85]. Since the construction of a premetastatic niche is an essential early step required for initiated cells to survive and evolve [86], it could be speculated that stem cells may contribute to the construction of premetastatic niches, at least in part, by secreting EVs. This could be supported through the observation of Grange et al., whereby interactions between endothelial cells and CSCs induced phenotypical changes in MSCs and promoted the formation of lung premetastatic niches through the release of EVs [38].

#### 5.1.1. Fibroblastic Differentiation and Stroma

Tumor cells have the ability to efficiently “educate” MSCs to induce changes in the local microenvironment through the release of EVs. For instance, tumor cell-derived EVs have been observed to propagate the construction of the tumor stroma through fibroblastic differentiation of MSCs [87]. Similarly, EVs from ovarian cancer cells have also been reported to induce adipose stromal cells (ASCs) to acquire the characteristics of tumor-supporting myofibroblasts [88]. A similar phenotypic switch was also noted in prostate cancer (PC) EVs whereby they influenced the differentiation of MSCs into proangiogenic and proinvasive myofibroblasts that was associated with disease promotion [89]. In another study, EVs from PC cells induced phenotypic transformation in PC patient ASCs to form prostate-like neoplastic lesions [90]. On top of that, tumor derived EVs can initiate phenotypic differentiation of MSCs into cancer-associated fibroblasts (CAFs) through signaling pathways [91]. These activated CAFs influence tumor progression through the secretion of metalloproteinases-rich EVs that promote cell motility and activate RhoA and Notch signaling in cancer cells [92]. Moreover, these EVs also play a role in chemoresistance and tumor relapse by promoting clonogenicity and growth of CSCs which are inherently resistant to cell death [93].

#### 5.1.2. Crosstalk among Stromal Elements

EV-mediated dynamic crosstalk within the stroma could relocalize the oncogenic factors that can generate a tumor-initiating niche. This can be speculated from the fact that carcinogenesis involves the relocalization of CAFs to the tumor site therefore sustaining metastasis [94]. The general involvement of EVs in intercellular communication suggests that they may also have a role in information exchange within stem cell hierarchies, whereby cancer stemlike cells may transmit signals to their stroma via EVs. Undeniably, it is well known that ESC-EVs transfer mRNAs, miRNAs, and proteins to recipient cells and therefore are important mediators of crosstalk within stem cell niches [95].

Stromal cell-derived EVs can interact with cancer cells and exchange oncogenic signatures present in tumor-associated stroma. For example, intercellular communication mediated by fibroblast-secreted EVs promotes cancer cell motility via autocrine Wnt-planar cell polarity signaling pathway to drive invasive activities [43]. Furthermore, the Wnt3a has been shown to be exported via EVs to neighboring cells which in turn modulates the population equilibrium in the tumor towards progression [96]. Such features are expected to be established very early during tumorigenesis; however, the prolonged intercellular communication could eventually be sustained and ultimately aggravate tumor growth. It could also be anticipated that the tumor microenvironment may host nontumorigenic multipotent stem cells that could likewise support the biological activity of tumor-initiating cells through the release of EVs.

#### 5.1.3. Endothelial Cell Growth and Angiogenesis

Angiogenesis is one of the underlying hallmarks of a developing tumor microenvironment. It is activated by proangiogenic factors such as VEGF thereby promoting tumor growth, invasion, and metastases. In response to hypoxia (a hallmark of developing tumors), MSCs increase EV production, which in turn promotes angiogenesis [113] and endothelial cell growth* in vitro* [114]. This proangiogenic property of stem cell-secreted EVs is reported to be linked with the activation of the ERK signaling pathway therefore increasing the expression of VEGF in tumor cells [68]. Furthermore, AT-MSC derived EVs have also been implicated to influence AT-MSC induced angiogenesis. Interestingly, the platelet-derived growth factor (PDGF) was identified to elicit this effect by stimulating AT-MSCs to release EVs with an enhanced proangiogenic potential [115]. In addition, AT-MSC derived EVs have been shown to interact with endothelial cells as well, thereby stimulating proangiogenic activity in the tumor microenvironment, possibly through the exchange of angiogenic growth factors [116].

EV-assisted targeting of key regulatory signaling pathways in the tumor microenvironment such as Wnt, Hedgehog, and angiogenic pathways (i.e., VEGF) could inhibit tumor growth. A recent study by Gernapudi et al. showed that a critical signaling axis, miR-140/SOX2/SOX9, which regulates differentiation, stemness, and migration in the tumor microenvironment, could be targeted to obstruct tumor progression [108]. Likewise, EV-assisted targeting of the VEGF pathway in the tumor microenvironment could exert antiangiogenic effects and inhibit tumor growth [74]. However, monitoring a complex stromal network in a dynamic tumor microenvironment has been and still remains technically challenging.

**Figure 2:**
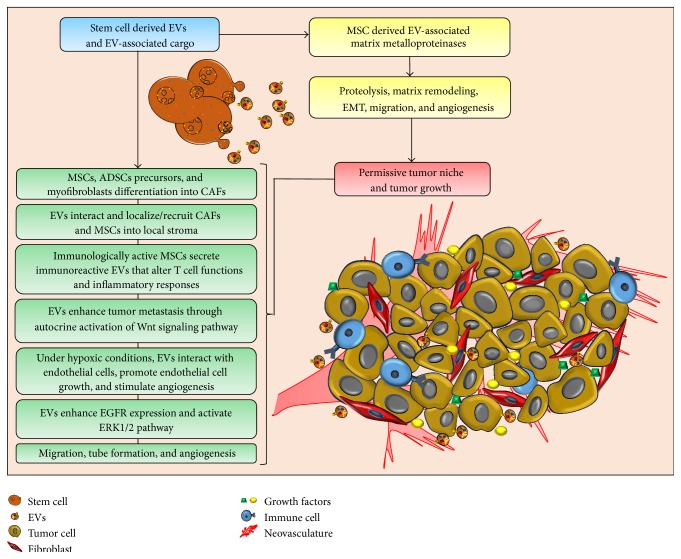
Contribution of stem cell-derived EVs in the construction of the tumor microenvironment. Stem cell-derived EVs influence the presence of cancer-associated fibroblasts (CAFs), inflammatory immune cells, metalloproteinases, angiogenic growth factors, and regulatory RNAs, which shape the tumor microenvironment. Extracellular matrix (ECM) remodeling, endothelial cell growth, cell migration, and angiogenesis generate a permissive tumor niche.

In summary, EVs secreted from cancer cells educate MSCs to undergo neoplastic transformation into tumor-associated fibroblasts in local stroma. In addition, EV-mediated dynamic interactions amongst stromal elements and the concomitant recruitment of oncogenic CAFs, growth factors, immune molecules, and several other related mechanisms shape a tumor niche capable of development and growth (Figure 2).

## 6. Stem Cell-EVs: Emerging Factors in Immunomodulation and Immunotherapy

There is increasing evidence implicating EVs to play a critical role in immune regulatory mechanisms comprising of both immunoactive and suppressive activities [117–119]. Conversely, studies elucidating the role of EVs from immunologically active stem cells are seldom reported in the scientific literature, possibly because their role as potential factors in immunomodulation is still novel. MSCs have been shown to secrete immunologically active EVs, which induce higher expression of anti-inflammatory transcripts such as interleukin 10 (IL10) and transforming growth factor *β*1 (TGF *β*1) and attenuated levels of proinflammatory transcripts such as IL1B, IL6, tumor necrosis factor *α* (TNF*α*), and IL12P40 [120]. Furthermore, infusion of MSC-EVs significantly enhanced the survival and delayed the rejection of allogenic skin graft in mice with a corresponding increase in regulatory T cells (Tregs) therefore indicating some role of EVs in immunomodulation [120].

Islet derived MSCs have been reported to release immunostimulatory EVs that could activate autoreactive B and T cells in nonobese diabetic (NOD) mice. Furthermore, immunization with EVs also promotes the expansion of transferred diabetogenic T cells and accelerates the effector T cell-mediated destruction of islets suggesting that stem cell-EVs act as autoantigen carriers with the potential to trigger autoimmune responses [97]. On the other hand, human adipose MSC derived EVs have been reported to exert inhibitory effects on the differentiation and activation of T cells followed by reduced T cell proliferation and IFN-*γ* release, therefore suggesting MSC-EVs to play a potential therapeutic role in the treatment of inflammation-related diseases [121]. Furthermore, EVs from immune cells can also have an effect on MSC cells. For instance, Ekström et al. showed that monocyte-derived EVs can induce increased gene expression of osteogenic markers in human MSCs, indicating that monocyte-derived EVs play a role in bone healing [122].

EVs from acute myeloid leukemia- (AML-) blasts that are positive for the hematopoietic progenitor cell antigen CD34 have been reported to be biologically active. Upon coincubation with natural killer (NK) cells, these AML-blast EVs efficiently downregulated the expression of surface NKG2D (an activator of NK cells) indicating a possible function in mediating immunosuppression in AML [123]. In addition, immunocaptured blast-derived EVs could also serve as biomarkers for AML during the course of therapy as a measure of disease progression or response to therapy.

Using a model of human-into-mouse xenogeneic graft-versus-host disease (x-GVHD), BMSCs were shown to play a role in immunomodulation, mediated by human CD4+ Th1 cells [124]. Notably, recipients treated with BMSCs had elevated levels of exosomes expressing CD73 in the serum that promoted the accumulation of adenosine* ex vivo*. Furthermore, on inhibiting the adenosine A2A receptor, the immunomodulatory property of BMSCs was abrogated, therefore implying a possible role of BMSC-EVs in an adenosine based immunosuppressive mechanism mediated in a paracrine fashion [124]. Recently, MSC-EVs were tested in a human patient to treat GVHD, whereby they were shown to be well tolerated [125]. Interestingly, the EV based treatment resulted in a reduced proinflammatory cytokine response to the patient's peripheral blood mononucleated cells (PBMCs)* in vitro* and the clinical GVHD symptoms improved significantly shortly after the start of the EV therapy [125].

Although initial findings ascertain that EVs could be a promising therapeutic source for the treatment of immune-inflammatory diseases [126, 127], translating this technology into EV-mediated stem cell therapy to achieve promising outcomes with minimized toxic effects and safety risks still needs to be explored.

## 7. Stem Cell-EVs and Tissue Repair

The therapeutic applications of stem cells for the treatment of various injuries and diseases have received considerable attention in recent years. However, investigations into the possibility of utilizing stem cell-EVs in regenerative medicine are still in their early stages. Recently, it has come to light that stem cells, particularly MSCs, use EVs for tissue repair and regeneration through the transfer of transcription factors, anti-inflammatory factors, and growth factors to target cells in a paracrine fashion. In this section, we discuss the regenerative abilities of MSC-EVs to recover vascular functions, repair injuries, and restore tissue homeostasis.

### 7.1. Stem Cell-EVs: Allies in the Fight against Cardiovascular Diseases

Stem cells have been suggested as an ideal novel therapeutic approach against cardiovascular diseases. However, an important feature of this approach is that EVs subsequently released from stem cells are a newly discovered source of cardiovascular protection. Improved heart function and vessel formation have been attributed to EVs released from stem cells, largely due to their anti-inflammatory and antiapoptotic effects (discussed below). Moreover, their effects can equally be extended towards neovascularization and cardiac regeneration.

Stem cell-EVs participate in the suppression of inflammatory responses in order to enhance cardiac functions. For instance, MSC-EVs mediate cytoprotective actions in a model of pulmonary hypertension by suppressing hyperproliferative pathways such as the signal transducer and activator of transcription 3 (STAT3) pathway as well as upregulating miRNAs that are downregulated during pulmonary hypertension [128]. Moreover, MSC-EVs moderate inflammatory reactions and rapidly ameliorate myocardial functions during ischemia/reperfusion injury, possibly by restoring bioenergetics, activating PI3K/Akt pathway, and triggering prosurvival signaling [129].

Cardiomyocyte progenitor cells (CPCs) have been shown to stimulate the migration of endothelial cells as well as promoting neovascularization that can potentially improve heart function [130]. Recently, EVs secreted by human CPCs have also been implicated with similar cardioenhancing functions mainly through inhibiting apoptosis, promoting tube formation, and initiating angiogenesis in cardiomyocytic cells [111]. Furthermore, MSC derived EVs also protect cardiac tissue from ischemic injury primarily by enhancing angiogenesis, blood flow recovery, and reducing infarct size [131]. After myocardial infarctions, they are linked with promoting recovery of cardiac functions via the Akt signaling pathway [132].

Ischemic preconditioning can potentiate the protective effects of MSCs during ischemic cardiomyocytes (CMs) injury. This is mainly achieved through the secretion of EVs that deliver miRNAs with antiapoptotic effects [109]. Furthermore, EVs released by the heart after ischemic preconditioning also contribute towards cardioprotection [133]. Interestingly, heat shock proteins have been shown to improve stem cell survival in an ischemic environment. This therefore promotes a prosurvival phenotype in CMs through heat shock factor-1 (HSF-1) and miR-34a interaction that is found to be mediated by EVs [134]. Other than proteins, MSCs are also capable of transferring antiapoptotic miRNAs to CMs via EVs that transcriptionally repress the expression of apoptotic genes, activate cell survival signaling pathway, and ensure cardioprotection [110, 135]. Such delivery of miRNAs could potentiate antiapoptotic effect of MSCs by inhibiting PUMA (p53-upregulated modulator of apoptosis) to promote cardiomyocyte survival [110].

Injured epithelial cells produce TGF-*β*1-containing EVs with defined genetic information able to activate fibroblasts, which initiate tissue regenerative responses and fibrosis [98]. Cardiac fibrosis is antagonized by stem cell-secreted EVs, exhibiting a substantial protection against myocardial infarction [109]. Furthermore, MSC-EVs have a replacement or cytoprotective effect, thus demonstrating potential therapeutic use in stem cell transplantation for the treatment of pulmonary fibrosis [136]. Notably, ESC-derived EVs can also effectively augment cardiac function in infarcted hearts through enhanced neovascularization, cardiomyocyte survival, and reduced fibrosis after infarction. The underlying basis for this beneficial effect was linked to the delivery of ESC specific miR-294 to CPCs promoting increased survival, cell cycle progression, and proliferation [112]. In addition, CD34+ stem cell-EVs also mediate the transfer of angiogenic factors to the surrounding cells, which may be beneficial in achieving functional recovery after ischemic injury [102]. Therefore, stem cell-EVs from various different tissue locations with their above mentioned healing properties have the potential to act as significant therapeutic agents in the fight against cardiovascular diseases.

#### 7.1.1. Protective and Therapeutic Role against Kidney Injury

MSCs have been implicated in protecting against renal damage through their paracrine effects. By use of a genetic fate-mapping technique, it has been shown that the recovery from acute kidney injury (AKI) depends on the intrinsic regenerative ability of tubular epithelial cells that undergo mesenchymal dedifferentiation and reentry into cell cycle [137]. Experiments performed by Bi et al. show that MSC conditioned medium has the ability to mimic the effects of MSC cells therefore indicating a paracrine form of action [138].

Recent evidence has also demonstrated that MSC-EVs could confer the paracrine actions of their parent cells and therefore contribute towards renal protection as well. For instance, Bruno et al. observed that human MSC-EVs induce recovery in a murine model of AKI in a manner comparable to that of the cell of origin [139]. Furthermore, evidence of mRNAs carried by EVs being translated into proteins has also come to light in both* in vitro* and* in vivo* conditions [139]. For example, Tomasoni et al. reported that MSC-EVs mediated the transfer of human IGF-1R mRNA to murine proximal tubular cells, which was subsequently followed by the enhancement of cell sensitivity to the regenerative actions of IGF-1 [100]. EVs from human umbilical cord-derived MSCs (hUC-MSCs) have also been demonstrated to trigger the ERK 1/2 pathway thereby inducing proliferation of tubular cells and protection against cisplatin-induced apoptosis [140].

EVs from human adult MSCs exhibit protective properties against AKI, largely by inhibiting apoptosis and stimulating proliferation of tubular epithelial cells [141]. This was further confirmed by Bruno et al., who showed, in a cisplatin-induced lethal model of AKI, that MSC-EVs enhanced the survival of mice through the upregulation of antiapoptotic genes and downregulation of proapoptotic genes [142]. This increased expression was attributed partly to EV-mediated miRNA transfer and in part due to the EV-triggered miRNA transcription [143]. Moreover, miRNA-depleted EVs released by Drosha knockdown MSCs failed to protect the kidney from acute injury [144], indicating that miRNA content of EVs is essential for their biological activity.

Using a mouse remnant kidney model, it was shown that BMSC-EVs could have protective effects against renal injury [145]. Furthermore, endothelial progenitor cell-derived EVs exert a protective effect against mesangial cell injury during Thy1.1 glomerulonephritis [146], and HLSC-derived EVs have the potential to influence renal function and morphology, in a manner comparable to the cells of origin [147]. Administration of MSC-EVs might also remarkably protect mice against renal failure, probably by transferring selective patterns of miRNAs from MSC-EVs to target cells and protection against EMT for restoration [148]. Hence, these studies concur on the therapeutic ability of stem cell-EVs and their potential to be applied as treatment for renal diseases in the future.

#### 7.1.2. Recovering Lung and Liver Injuries

EVs from lung cells have been shown to induce the expression of mRNAs coding for lung specific proteins such as Clara cell protein and aquaporin-5 and A-D surfactants in bone marrow cells [149]. Furthermore, this phenomenon is enhanced significantly during lung injury [149].

Acute and chronic lung injuries could in theory be recovered through the paracrine actions of MSC-EVs which confer a stem cell-like phenotype in injured cells for the activation of self-regenerative programs [99]. Moreover, ischemic preconditioning can also potentiate the protective effects of MSCs against endotoxin-induced acute lung injury through the secretion of EVs [150].

HLSC-EVs may also have a role in accelerating the morphological and functional recovery of injured cells during liver damage, mainly through horizontal transfer of specific mRNA subsets to recipient cells [151]. Recently, it has been reported that transplantation of EVs released from hUC-MSCs could reduce the surface fibrous capsules, soften their textures, and alleviate hepatic inflammation in fibrotic liver [103]. Moreover, it was observed that human liver cells undergo typical EMT after induction with recombinant human TGF-*β*1, whereas MSC-EV treatment reverses the expression of EMT-associated markers [103], indicating that MSC-EVs could prevent liver fibrosis and ameliorate hepatocyte protection through EMT inhibition. Furthermore, MSC-EVs appear to promote hepatic regeneration following drug-induced liver injury, mainly through the activation of proliferative and regenerative responses [152]. This therefore demonstrates the ability of EVs to act as functional messengers of stem cells.

#### 7.1.3. Stem Cell-EVs and Neuroprotection

There are several studies that have recently come to light that associate MSC-EVs with a neuroprotective role along with stem cells. For instance, EV-mediated transfer of miRNAs from MSCs promotes neural plasticity and functional recovery of neurons and astrocytes after treatment for stroke in rats [107]. Furthermore, EV-mediated delivery of miRNAs enhances the expression of glutamate transporters, suggesting an efficient route of therapeutic miRNA delivery to the brain. A recent study by Raisi et al. reported that EVs produced from anti-inflammatory MSCs enhanced sciatic nerve regeneration in rats which could be potentially applied in peripheral nerve cell therapy [153]. In addition, EVs from embryonic cerebrospinal fluid have been reported to carry evolutionarily conserved proteins and miRNAs, which promote neural stem cell proliferation [154].

The protective effects of MSC-EVs were recently observed against glutamate-induced excitotoxicity in rat pheochromocytoma via a PI3K/Akt dependent pathway [104]. Investigation of a possible underlying mechanism for this protection revealed that MSC-EVs were responsible for downregulating Bax expression accompanied by reduced cleavage of caspase-3, upregulation of Bcl-2 expression, and Akt phosphorylation in glutamate-treated cells, therefore providing a prosurvival fate for affected cells [104]. Transplantation of MSC-EVs could therefore be a potential strategy to treat neuronal diseases involving excitotoxicity.

MSC-EVs may also have effects on neurovascular plasticity during traumatic brain injury by inducing endogenous angiogenesis and neurogenesis and reducing neuronal inflammation [155, 156]. Furthermore, a study by Katsuda et al. has reported the role of human AT-MSCs derived EVs as a novel therapeutic approach for Alzheimer's disease (AD) as they were found to be loaded with enzymatically active neprilysin (NEP) that potentially contributes to amyloid-*β* clearance in the brain [105]. These observations and others therefore support the potential of stem cell-EVsas a means of cell-freetherapyagainst cerebral injuries and neurologicaldiseases.

**Figure 3:**
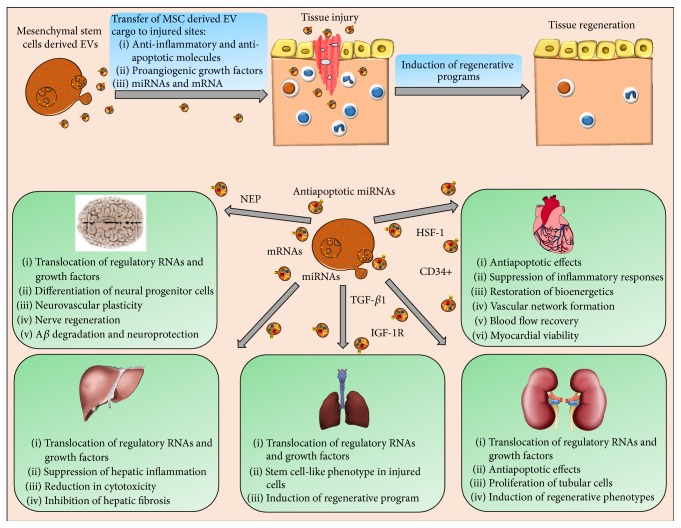
Schematic representation of the regenerative effects of stem cell-derived EVs. MSCs use EVs to ameliorate tissue damage through translocating growth factors, anti-inflammatory, antiapoptotic, and proangiogenic molecules, to sites of injury where they induce and regulate regenerative phenotypes.

In summary, stem cells induce regenerative programs in injured tissues of different organs through EV-mediated transfer of anti-inflammatory miRNAs and growth factors. These, in turn, ameliorate the damage (Figure 3) by activating different signaling pathways, which have an antiapoptotic and angiogenic effect therefore significantly restoring the bioenergetics, improving blood flow recovery, and inducing stemlike phenotypes in injured organs.

The ability of EVs to mediate bidirectional intercellular communication suggests that they could not only send information from stem cells to injured cells but also communicate from injured cells back to stem cells. In this regard, the future clinical contributions of EVs could therefore be (1) translocation of regenerative signatures at injured sites to trigger repair process, (2) transmission of signals related to tissue injury towards stem cells, to generate more progeny for maintaining stem cell equilibrium and continuous supplies to injured sites, and (3) EVs from injured tissues that could also stimulate stem cells to acquire features of the injured cells.

## 8. Concluding Remarks and Future Perspective

Stem cell-EVs provide a new interpretation of stem cell plasticity that is still a hotly debated topic [157]. EV-mediated bidirectional exchange of information between progenitor cells and tissue differentiated cells leads to phenotypic changes that provide evidence of previously uncertain cell plasticity. This could be beneficial to clinical implications of EVs for future regenerative medicine.

Elucidating the contribution of stem cell-EVs in premetastatic niche formation and biological characteristics of the cancerous stromal elements may offer novel targeting opportunities and have prognostic and predictive values. However, monitoring the target complex tumor microenvironment remains a technical challenge.

Moreover, the immunomodulatory features of MSC-EVs could be extended in the treatment of immune diseases, reducing potential safety risks originated from conventional immunotherapy such as susceptibility to infection, immune dysfunction, autoimmune responses, and/or increased risk of developing cancer. Conceivably, EVs from immunoreactive MSCs could be novel therapeutic tools against inflammation-associated diseases [125].

Despite the improvements of surgical procedures in organ transplantation and cell-based therapies in the last decade, current methods present potential complications (i.e., toxicity and organ rejection), which remains dismal for improving long-term survival and reducing mortality. It is therefore necessary to look for alternative and more reliable procedures, which could potentially minimize associated risk factors. In this regard, EV based cell-freetherapy could be a preferred option as it would improve patients' outcome considerably, by reducing the complications associated with cell-based treatments. For instance, ESC-EVs provide a novel cell-free system that uses the immense regenerative power of ES cells while avoiding the risks associated with direct ES or ES-derived cell transplantation and risk of teratomas [112]. Furthermore, EVs have the ability to extend the therapeutic effects and functions of stem cells in both a local and systemic environment. Hence, the administration of stem cell-derived EVs would reduce potential safety risks associated with cellular therapy and/or transplantation surgery.

However, further studies are needed to define whether EVs permanently or transiently change the “genetic fingerprint” of an injured target organ and the consequences of it. Preliminary studies on acute kidney injury indicate a restoration of a persistently normal renal molecular pattern after EV treatment [144]. However, the effect may be dose dependent and increasing doses of EVs may produce unwanted effects as we observed in preliminary experiments with adipose-derived MSCs.

As MSCs have the potential to differentiate into other types of viable replicating cells, replacing their transplantation with administration of MSC-EVs (which mimic similar functions as MSCs) would reduce this possibility and maintain the therapeutic benefits at the same time. However, the mobilization of EVs and homing to specific tissues are still a major concern. Given that EVs express specific membrane receptors and proteins specific to certain cell types [158], it may be possible to identify a potential mechanism to direct EVs as well as stem cells to a particular tissue. Receptor-ligand mediated interactions and homing could also facilitate the cellular uptake of EVs and their associated cargo by recipient injured cells. Furthermore, as a reparatory response, the mobilization of EVs to injured zones could be a result of dynamic interactions among stromal elements. It is quite possible that, in response to an injury or transplant, the enhanced release of EVs could create a gradient that may educate them to mobilize to the site of injury.

Since EVs are more suitable for monitored manufacturing processes, they could be engineered to express and deliver regenerative signatures to target sites. However, a potential challenge is the lack of standardization of the existing technologies to isolate and characterize EVs for their effective therapeutic utility. Therefore, a combination of different high throughput methods could overcome the potential limitations related to EV detection and characterization [5]. Particularly, the technologies for the isolation and characterization of stem cell-EVs must be improved and optimized [159], ensuring the purity of EVs that is critical for developing cell-free therapeutic strategies using stem cell-EVs in the future.

## Figures and Tables

**Table 1:** Stem cell signatures expressed and secreted in EVs.

Components	Stem cell type/source	Reference
Nanog, Oct-4, HoxB4 and Rex-1	ESCs	[37]
CD105	CSCs, MSCs	[38, 97]
Prominin-1/CD133	HPCs, melanocytes	[39, 40]
KIT	Mast/stem cells	[42]
WNT	Fibroblasts, DLBCL	[43, 44, 96]
*β*-catenin	HEK	[45]
Stem-cell antigen-1	MSCs	[97]
TGF-*β*1	Fibroblasts, epithelial cells	[98]
TIA, TIAR, HuR, Stau1, Stau2, RPS29, and Ago2	MSCs	[54]
KGF	MSCs	[99]
*Oct4* and *Sox2* mRNAs	ESCs	[62]
IGF-1R mRNA	MSCs	[100]
DEFA3, HBB, ITGA2B, and ITGB3 mRNAs	HPCs	[101]
VEGF	MSCs	[68]
PDGFR-*β*, TIMP 1 and 2, sphingomyelin, lactic acid, and glutamic acid	MSCs	[69]
CD34	MSCs	[102]
E-cadherin	MSCs	[103]
Bcl-2	MSCs	[104]
NEP	MSCs	[105]

ESCs: embryonic stem cells, CSCs: cancer stem cells, MSC: mesenchymal stem cells, HPCs: haematopoietic precursor cells, DLBCL: diffuse large B-cell lymphoma, HEK: human embryonic kidney cells, KIT: mast/stem cell growth factor receptor, IGF-1R: growth factor receptor, TGF-*β*1: transforming growth factor beta 1, KGF: keratinocyte growth factor, ITGA; integrin alpha, VEGF: vascular endothelial growth factor, PDGFR-*β*: platelet-derived growth factor receptor beta, TIMP: tissue inhibitor of metalloproteinase, and NEP: neprilysin.

**Table 2:** Selectively enriched regulatory miRNAs from stem cell-derived EVs.

miRNAs	Stem cell type/source	Reference
miR-199b, miR-218, miR-148a, miR-135b, and miR-221	MSCs	[106]
miR-223, miR-564, miR-451, and miR-142-3p	MSCs	[54]
miRNAs of 290 cluster	ESCs	[62]
let-7 miRNA family	MSCs	[64]
miR-133b	MSCs	[107]
miR-15a	MSCs	[66]
miR148a, miR532-5p, miR378, and let-7f	MSCs	[67]
miRNA-21, 34a	MSCs	[69]
miR-23b	MSCs	[73]
miR-16	MSCs	[74]
miR-140	Preadipocytes	[108]
miR-22	MSCs	[109]
miR-221	MSCs	[70, 110]
miR-210, miR-132, and miR-146a-3p	CPCs	[111]
miR-294	ESCs	[112]
